# Fate decisions of breast cancer stem cells in cancer progression

**DOI:** 10.3389/fonc.2022.968306

**Published:** 2022-08-15

**Authors:** Hui Xu, Fengxia Zhang, Xiaokang Gao, Qiwang Zhou, Linhai Zhu

**Affiliations:** Department of General Surgery, The Affiliated Hospital of Yangzhou University, Yangzhou, China

**Keywords:** breast cancer stem cells, breast cancer, heterogeneity, tumor microenvironment, transcription factors, non-coding RNAs

## Abstract

Breast cancer has a marked recurrence and metastatic trait and is one of the most prevalent malignancies affecting women’s health worldwide. Tumor initiation and progression begin after the cell goes from a quiescent to an activated state and requires different mechanisms to act in concert to regulate t a specific set of spectral genes for expression. Cancer stem cells (CSCs) have been proven to initiate and drive tumorigenesis due to their capability of self-renew and differentiate. In addition, CSCs are believed to be capable of causing resistance to anti-tumor drugs, recurrence and metastasis. Therefore, exploring the origin, regulatory mechanisms and ultimate fate decision of CSCs in breast cancer outcomes has far-reaching clinical implications for the development of breast cancer stem cell (BCSC)-targeted therapeutic strategies. In this review, we will highlight the contribution of BCSCs to breast cancer and explore the internal and external factors that regulate the fate of BCSCs.

## Introduction

Breast cancer is the second major risk of cancer death in women (1). At present, surgical resection is the preferred treatment for breast cancer, including breast-conserving surgery (BCS) and mastectomy, and a series of comprehensive treatment measures such as chemotherapy, radiao-therapy, hormone therapy and other novel therapies are combined according to clinical-pathology. Despite increasingly accessible systems for early diagnosis and therapy of breast cancer, it remains the most prevalent of female malignancies in terms of mortality. Recurrence and metastasis are the main reason for the increase in mortality (2–4). Most breast cancer patients express receptors for estrogen (ER) and progesterone (PR) and therefore respond to hormone therapy or aromatase inhibitors. However, triple negative breast cancer (TNBC) lacks the expression of ER, PR and human epidermal growth factor receptor-2 (HER-2) (5). Breast cancer contains a heterogeneous cell population and is divided into for major molecular subtypes according to genetic expression, including luminal A, luminal B, HER2-enriched, and triple-negative (6, 7). Certain subtypes are prone to drug resistance, resulting in limited treatment efficacy, which poses a significant challenge to clinical cure and survival of breast cancer patients.

BCSCs are a class of cells with the ability to continuously self-renew, proliferate indefinitely and differentiate in multiple directions, and possess multiple drug-resistant molecules that are the main cause of drug resistance in breast cancer (8–10). There are two hypotheses on the origin of BCSCs: one is that BCSCs originate from adult stem cells and can acquire malignant behaviors by changing their genetic characteristics; the other is that BCSCs are transformed by early progenitor cells that have acquired the ability to self-renew (11, 12). The concept of BCSCs has been further developed to be involved in mediating tumor heterogeneity, with the ability to clonally regenerate tumors after seemingly successful treatment, and is of profound importance in understanding and treating hierarchically organized breast cancer (13). Therefore, further understanding of the fate decisions of BCSCs, identifying significant roles in tumor recurrence, metastasis and drug resistance, and developing therapeutic strategies to target BCSCs are of great clinical significance for the treatment of breast cancer. Hence, we outline the hierarchy of BCSCs in the origin of breast cancer and their role in tumor heterogeneity, recurrence, metastasis and drug resistance, in conjunction with a discussion of the potential of BCSCs as therapeutic targets to provide clinicians with new strategies to improve breast cancer treatment.

## Unraveling the routes of mammary stem cell differentiation

A highly dynamic organ that produces and secretes milk to nourish offspring, the mammary gland undergoes multiple phases of remodeling throughout a female’s life and consists of two main parts: the parenchyma and surrounding stroma. The parenchyma contains mainly epithelial cells, glandular cells and myoepithelial cells: the epithelial cells are located in the inner layer of the milk ducts; the glandular cells form the alveoli, whose main function during lactation is to secrete milk; and the myoepithelial cells form the basement membrane, which usually surrounds or separates the epithelial cells from the glandular cells (14, 15). The proliferation and differentiation of the mammary gland is regulated by hormones and growth factors, for example estradiol, progesterone and prolactin. According to the characteristics of mammary gland development, it can be roughly divided into six developmental stages: embryonic stage, birth to early sexual maturity, sexual maturity, pregnancy, lactation and involution, as well as each estrous/menstrual cycle, both local and systemic stimuli can set off the mammary cell expansions and/or differentiation (16).

The mammary gland shows such obvious periodicity because a hierarchical array of mammary stem cells (MaSCs) and progenitor cells (PCs) are located in the organ, which maintain the homeostasis under physiological conditions (17). The differentiation of MaSCs is a two-step journey, consisting of cell lineage determination (from MaSCs to PCs of a specific lineage) and maturation (from PCs to particular cell types). These cells can yield all the mature cell types in the mammary gland, including ductal, alveolar and myoepithelial, and the primary outgrowths contain daughter cells that have the same regenerative capacity as the original stem cells (18). Thus, these cells have the dual hallmarks of stem cells, multidirectional differentiation and the ability to self-renew. Stem cell fate decisions, which begin after the cells differentiate from a quiescent to an activated state, require different mechanisms to coordinate and regulate the expression of a specific set of lineage genes. The presence of stem cells is necessary for the regeneration of the mammary gland and is important for studying the mechanisms of organogenesis and cell differentiation, but abnormal differentiation and proliferation of stem cells can lead to occurrence of tumors.

## Stem cells as the cellular origin of breast cancer

**Figure 1:**
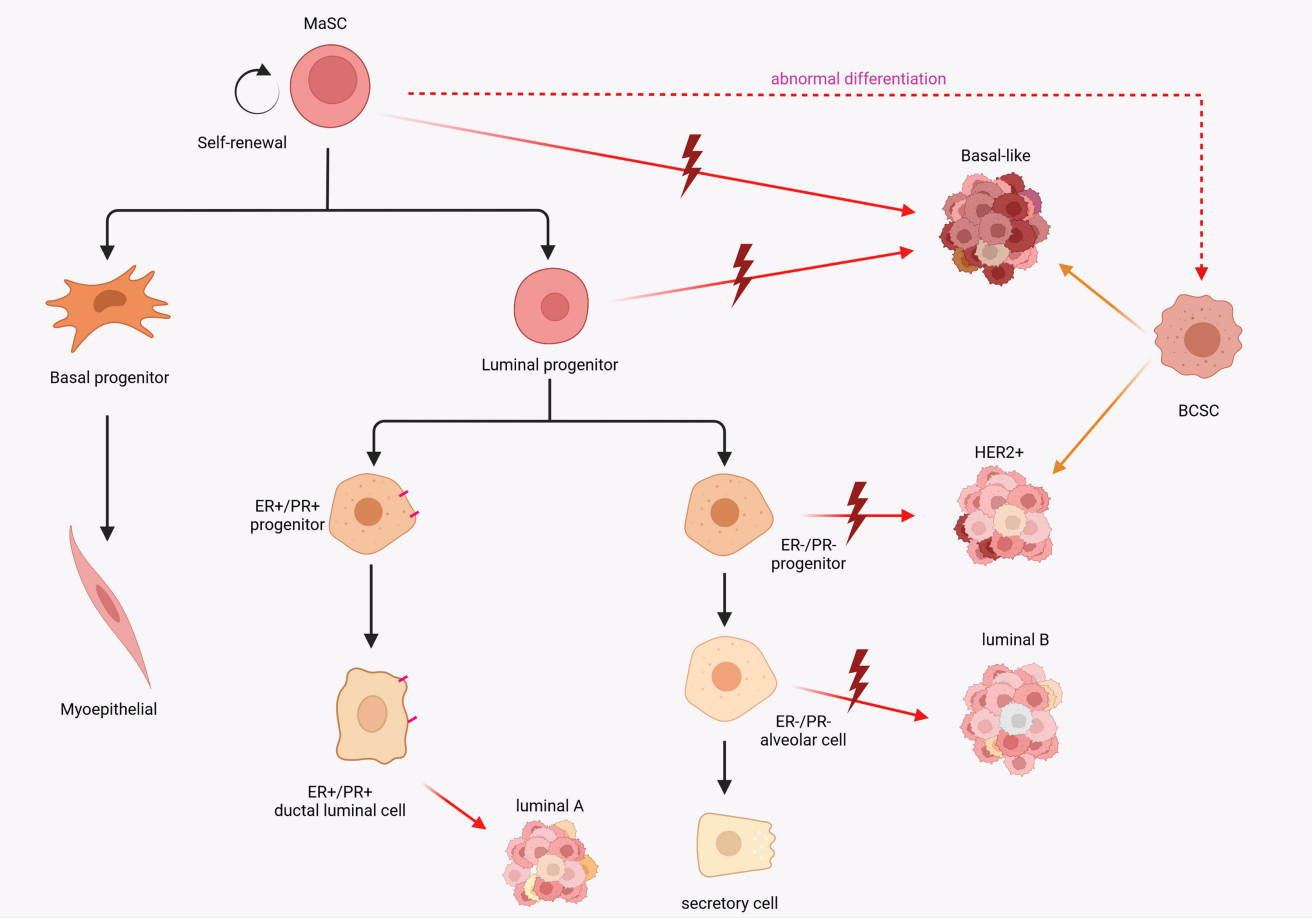
Schematic diagram of the potential relevance of breast epithelial cell hierarchy and breast cancer stem cell origin to breast cancer subtypes. MaSCs expose to mutations that cause abnormal differentiation and transformation into cancer stem cells. A comparison between specific molecular features of normal breast epithelial subpopulations and different breast cancer subtypes revealed that the tumor subtypes appeared to have similar differentiation characteristics to normal breast cells.

Unmasking the origins of breast cancer to be still a challenging and creative topic in the field of oncology research. The cellular origin of cancer continues to be an important scientific question. Two major models have been developed to describe the cellular origin of cancer. In the somatic mutation model, the stepwise accumulation of a series of independent mutations in differentiated cells promote the capability to gradually reprogram and obtain malignant genotypes (19, 20), while the second hypothesis involves mutations in stem or progenitor cells (21). It is inevitable that these two models will co-exist. About 5-10% of breast cancers are inherited susceptibility due to germline mutations, such as BRCA1 and BRCA2 (22–24). Using single-cell assays, in BRCA1 mutation breast cancer, basal-like breast cancer (ER^-^) and luminal breast cancer (ER^high^) respectively derive from luminal progenitors and mature luminal cells respectively (25). These discoveries indicate that breast cancers may be initiated by mutations in differentiated cells. Interestingly, it is evident that cancer cells and stem cells share many characteristics, including high proliferative capacity, longevity, pliancy and the activity of molecular pathways that regulate stem cells (26). Corinne A Boulanger1 and Gilbert H Smith’s inventive research in breast cancer was the first to demonstrate that the mammary epithelial stem cells were indeed responsible for the evolution of carcinogenesis in mature mammary gland and formed tumor stem cell populations (27). Increased expression of stemness-associated genes, such as pseudokinase Tribble 3 (TRIB3) (28), NOTCH1 (29) and SOX9 (30), is positively correlated with the development of breast cancer. Patients with TNBC tend to have a comparatively worse outcome than other subtypes, due to their inherently invasive trait and the lack of molecular targets for treatment (31). TNBC is often, but not always, a basal-like subtype and expresses basal like markers (K5, K14, ITGA6, P-cadherin and Id4) with the character of stemness (32, 33). Definitely, high expression of stem cell-related gene traits in the BCSC subpopulation were a potential predictor of worse prognosis (34, 35). Interestingly, it is hypothesized that the apparent heterogeneity within breast cancer tumors reflects the different mammary epithelial cells as the cellular origin and drivers of malignant transformation (36, 37). A comparison between specific molecular features of normal breast epithelial subpopulations and different breast cancer subtypes revealed that the tumor subtypes appeared to have similar differentiation characteristics to normal breast cells. The basal-like subtype expresses intracavitary progenitor cell markers. This appears to correlate with the basal MaSC molecular subtype and therefore intuitively indicates that MaSC are a potential cellular source of basal-like breast cancer. Correspondingly, the HER2, luminal A, and luminal B subtypes express luminal lineage markers (18) (**Figure 1**). Collectively, these discoveries provide several insights into the origin of breast cancer cells: 1. Is oncogene-induced transdifferentiation of mammary gland cell sufficient to explain the plasticity and heterogeneity observed in breast cancer? 2. Does the continuous differentiation of normal stem cells to replenish the pool of progenitor cells during the maintenance of mammary gland homeostasis contribute to tumorigenesis?

## Stem cell hierarchies in breast cancer

**Figure 2:**
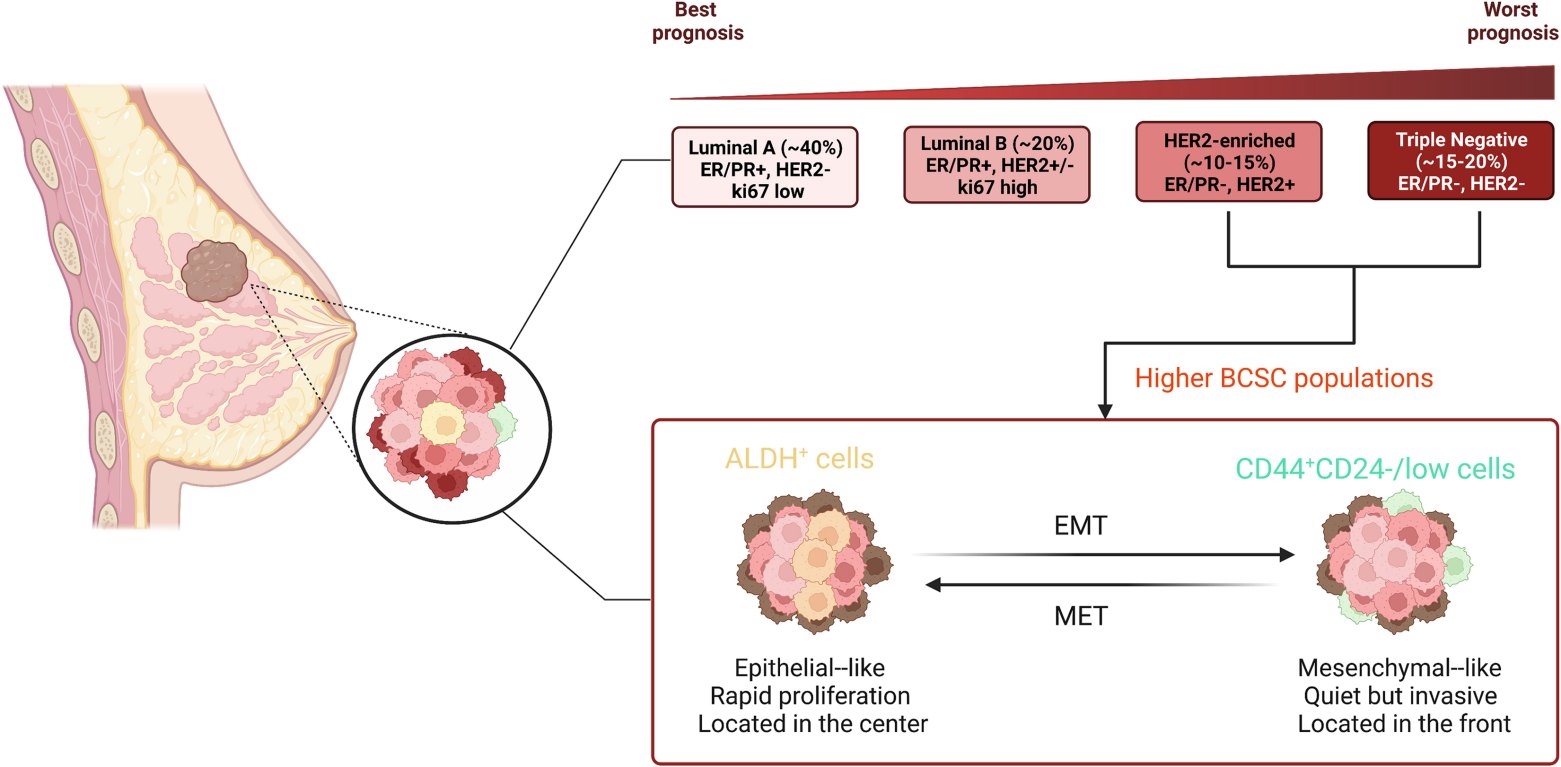
Different subtypes of breast cancer and distinct state of BCSCs. Breast cancer contains a heterogeneous cell population and is divided into four major molecular subtypes according to genetic expression, including luminal A, luminal B, HER2-enriched, and triple-negative. The typical molecular expression in each subtype is shown in the figure. There are two major distinct phenotypes of BCSCs: CD44^+^CD24^-/low^ and ALDH^+^. BCSCs (CD44^+^CD24^-^) are mesenchymal-like and predominantly quiescent and localized at the front of the tumor invasion, whereas epithelial-like BCSCs expressing ALDH are more proliferative and more centrally located.

During the last few decades, numerous studies have demonstrated that both tissue stem cells and CSCs can survive for long periods of time and have a great proliferative capacity, which means not only that they can accumulate many mutations, but also that they share the same capacity for reversibly entering a quiescent cell-cell state, multidirectional differentiation, an overlapping immunophenotype and gene regulatory networks (26). Through xenotransplant experiments, Al-Hajj together with colleagues presented directly the first investigated evidence for the presence of so-called BCSCs (CD44^+^CD24^-/low^Lineage^-^), which are located at the top of breast cancer with a hierarchical structure (38). Subsequently, Christophe Ginestier and colleagues raveled out that aldehyde dehydrogenase (ALDH) can act as a potential marker for BCSCs, these cells with the widest lineage differentiation potential and the greatest capacity for growth (39). Surprisingly, BCSCs (CD44^+^CD24^-^) are predominantly quiescent and localized at the front of the tumor invasion, whereas epithelial-like BCSCs expressing ALDH are more proliferative and more centrally located (40). High-throughput sequencing technologies developed in recent decades have enabled us to accumulate a wealth of relevant data on BCSC hierarchies and fate decisions. At the single-cell level, BCSCs showed high tumorigenesis and expressed stemness and EMT-related genes, in particular ZEB2, SOX2, ID1 and TWIST1 (41). Along with the ground, acquiring the properties of EMT allows the cells to be reprogrammed to a more stem-like state (42). Importantly, BCSCs, located at the top of the cancer hierarchy, are considered to be in line with their healthy MaSCs, and they can exacerbate breast cancer. More studies have confirmed that BCSCs expressing relevant cell surface markers have biological properties similar to MaSCs, including ALDH1^+^ (39), CK5^+^ (43), CD49f^+^ (44), ITGA6^+^ (45). These discoveries provide insightful evidence not only of the clinical relevance of BCSCs in breast cancer, but also indicate that breast cancer should be uniquely therapeutic according to their gene profile (**Figure 2**).

Noticeably, not all cancers are considered to be stringently hierarchical in organization. It is generally believed that the CSC lies at the top of the hierarchy, whereas in reality the CSC hypothesis is more complex than a simple linear model. Several discoveries have shown that some non-CSCs can lead to dedifferentiation through genetic mutation, and exhibit plasticity by reversibly transitioning between a stem and non-stem state (46). Importantly, the hierarchical heterogeneity is beyond genetic mutations and covers non-genetic characteristics with regard to epigenetic programs, immune characteristics, inflammatory states and microenvironmental composition. Lineage plasticity is important for the development of aggressive BLBC, transcription factor SOX9 can regulate cell phenotypic plasticity and breast cancer progression (30). Similarly, EMT-mediated phenotypic plasticity has important clinical implications for breast cancer progression and drug resistance (47). Similar to normal breast cells, BCSCs are able to respond to external or internal stimuli, debugging their phenotype and behavior *via* EMT, reversible quiescence and senescence or metabolic plasticity to counteract the stress of treatment. All of these characteristics contribute to the resistance to treatment of CSCs. For example, BCSCs have built-in mechanisms to promote phospholipid metabolism and the generation of free fatty acids, which activate the relevant signals and maintain stemness, thus contributing to chemo-resistance as cisplatin, doxorubicin, or tamoxifen. In this case, phospholipase A2 inhibitors, such as Giripladib, are required in combination to effectively eliminate BCSCs and inhibit tumorigenesis (48). Alexander Swarbrick and his colleagues demonstrated that stromal cues form CAFs, including FGF5 and fibrillar collagen, are capable of inducing and maintain a stem-like phenotype in TNBC cells by providing a supportive niche (49). Corporately, these evidences suggest that BCSCs are not in a specific-widespread phenotype but stay at a certain plasticity. In conclusion, the model of tumor origin and evolution is not limited to a single hierarchical level, but also needs to take fully into account the polyclonal heterogeneity that characterizes the successive interactions between different cell populations. Certainly, they are not mutually exclusive and there may be transitions between BCSC and BCSC-like states, and the concept of BCSC populations needs to be treated more dialectically, that is, there may be multiple BCSC populations of different subtypes.

## BCSCs as participant of breast cancer heterogeneity

Breast cancers show marked heterogeneity due to genomic, transcriptomic and microenvironmental differences, resulting in different phenotypes and variability in biological behavior (50). Due to inter-tumor heterogeneity, they can be classified into different types based on their morphology, molecular expression or genomic copy number patterns. There are currently two models that address the issue of heterogeneous origins: the clonal evolution model and the CSC model (37). These two hypotheses are not independent, but rather a coexisting, dynamic process that provides a theoretical basis for explaining inter-tumor heterogeneity and intrinsic differences in the regenerative capacity of breast cancer (51). The clonal evolutionary model assumes that any undifferentiated and differentiated cell is capable of accumulating mutations that lead to the creation of clonal populations of cells within a tumor, while individual tumor cells in a monoclonal clone share a degree of identical genetic variation, and different subpopulations of tumor cells have the ability to mutate individually during tumor evolution, thereby mediating the creation of tumor heterogeneity. The CSC model holds that a tumor actually consists of a cluster of stem cells, as well as cells that are unevenly differentiated, and can explain breast cancer tumorigenesis (52, 53). Typically, CSCs in tumors are genetically unstable, with multiple isoforms, and CSCs that survive adaptively in a clonal pool following altered microenvironmental niches and targeted therapeutic approaches, mediating the intar- and/or inter-tumor hierarchy and promoting malignant progression. In conclusion, further refinement and emphasis on the evolutionary and adaptive CSC dynamic concept is complementary to explain the possible causes of tumorigenesis, recurrence and metastasis. Therefore, eliminating the most diverse types of tumor cells, including BCSCs, is the most fundamental strategy for curing breast cancer.

## BCSC identity markers

The development of BCSC-specific biomarkers for breast cancer has expanded the understanding of heterogeneity and has been further validated in both *in vivo* and *in vitro* breast cancer models. These breast cancer stem cells represent only a small fraction of the cells within the tumor and are extracted by flow cytometry technique capable of identifying certain patterns of surface markers (54, 55). A growing number of studies have revealed and characterized BCSC markers, and these markers have been shown to identify different stem cell populations well. As mentioned above, CD44^+^CD24^-^ and ALDH^+^ are common molecular markers for BCSCs. Equally important, due to the highly heterogeneous character of breast cancer, in which more different phenotypes of BCSCs may exist, the discovery and identification of their biological functions could achieve a substantially more constructive reaction to anti-cancer therapy in the design of new drugs targeting BCSCs (**Table 1**).

**Table 1:** Principal BCSC identity markers.

Phenotypes	Sample sources	IsolationIdentification	Ref.
CD44^+^/CD24^−/low^	Human primary breast tumor Pleural Effusion Injections	FACS	(38)
ALDH^+^	Human breast tumors	FACS ALDH1 IF	(39)
CD133^+^	BRCA1^Δexon11^p53^+/-^ mouse mammary tumors	FACS CD133 IF	(56)
CD24^+^CD29^+^ and CD24^+^CD49f^+^	BRCA1-mutant mouse mammary tumors	FACS Tumorsphere	(57)
CD44^+^CD24^-^ESA^+^	Human SUM159, SUM1315 and MAD-MB-231 cell lines	FACS BrdU label	(58)
CD49f^+^EpCAM^+^	BRCA1-mutant human mammary tissues	FACS Microarray hybridizations	(59)
GD2^+^	Human breast tumor tissue and SUM-159, HS578T, MDA-MB-231 and MDA-MB-468 cell line	FACS Microarray analysis	(60)
CD90^hi^	Human MAD-MB-231 cell line	FACS CD90 IF	(61)
CD133^high^CXCR4^high^ -ALDH1^high^	Human breast tumor tissue with chemo-treated patients	Sphere-formation	(62)

## Regulatory mechanisms of BCSCs

The establishment of the BCSC theory provides the theoretical basis for explaining the hierarchy and heterogeneity of breast cancer. These fickle BCSC populations initiate and fuel tumor growth and are intimately associated with intrinsic treatment-resistant. BCSCs possess significant stemness and plasticity, and their fate decisions that extensive and complex regulatory mechanisms are required to coordinate and regulate the expression of specific lineages of genes, starting after the cell differentiates from a quiescent to an activated state. Here we focus on the contribution of transcriptional regulation, signaling pathway, epigenetic regulation, and post-transcriptional modifications that occur during this process.

## Transcription factors

Transcription factors (TFs), also known as trans-acting factors, are functional protein molecules that specifically bind to DNA and regulate gene transcription. Most TFs bind to DNA before forming dimers or multimers through protein-protein interactions. In addition to TFs that bind DNA directly, there are regulatory proteins that do not bind DNA directly, but rather bind DNA indirectly through protein-protein interactions, regulating gene transcription and thus forming expression regulatory complexes. The gene expression that defines the phenotype is highly coordinated. As a result, regulatory programs meticulously curated by crucial TFs have been posited to have a central function in the determination of cell fate.

Evidently, intratumoral hypoxia is a common manifestation in advanced cancers. In hypoxic breast cancer cells, HIFs activate the transcription of target genes that play important roles in tumor progression, metabolic reprogramming, motility and chemoresistance (63). Numerous studies have shown that the response of BCSCs to hypoxia requires HIFs to regulate and maintain the direct or indirect transcriptional regulation of BCSC stemness-related factors including NANOG, SOX2, and KLF4 (64, 65). In addition, HIF-1α maintains the onset of hypoxia-induced EMT and regulates the plasticity of BCSC (66, 67). Recently, researches showed that HIF-dependent ALKBH5 and S100A10 expression mediates the enrichment of BCSCs in the hypoxic tumor microenvironment (68, 69). Similarly, HIF-1 can directly activate calreticulin (CALR) transcription and facilitate breast cancer progression by promoting the BCSC phenotype in hypoxic (70). Collectively, these discoveries exhibit that hypoxia increases the percentage of BCSCs and governs their phenotypic transformation in a HIF-dependent manner.

Metastasis is the cause of up to 90% of cancer-related deaths, yet it continues to be the least known integral part of cancer pathogenesis. The most common sites of metastasis from breast cancer are bone, lung, brain and liver. Truncated glioma-associated oncogene homolog 1 (TGLI1) was found to transcriptionally activate the expression of CD44 and OCT4, contributing to BCSC renewal and thus promoting brain metastasis (71). Mechanistically, malignant progression in breast cancer is accompanied by an increase in the proportion of these BCSCs within the tumor and activation of the EMT (72, 73). EMT is a complex transdifferentiation program characterized by the loss of epithelial-specific features accompanied by the acquisition of mesenchymal phenotypes that fuels non-transformed cells and tumor cells to acquire stemness (74, 75). The loss of epithelial-specific features means that the tumor is more aggressive and has a poorer prognosis. Intrinsically, EMT-associated TFs (EMT-TFs) were crucial regulatory mechanism for tumor progression and metastasis including, Snail 2, Twist 1, Slug, SOX2/9 and Zeb1/2.

## Non-coding RNAs

In contrast to well-known molecular signaling pathways, the involvement of non-coding RNAs (ncRNAs) in CSC lineage commitment has only just been discovered. Based on their biological functions, ncRNAs are divided into two major categories: housekeeping ncRNAs and regulatory ncRNAs. Regulatory ncRNAs can be divided into short chain ncRNAs and long chain ncRNAs according to the sequence length. ncRNAs with short chains include microRNA (miRNA), small interfering RNA (siRNA), piRNA and transcription initiation RNA (tiRNA) have the characteristics of small molecule and high sequence conservation. Whole-genome sequencing revealed that ncRNAs comprise 98% of the human gene transcriptome and consist mainly of miRNAs and LncRNAs that do not have protein-coding functions (76). A variety of miRNAs and LncRNAs are responsible for the modulation of BCSCs.

## microRNAs

miRNAs are commonly expressed in organisms that are approximately 18-25 nucleotides in length and can complement the 3’-UTR of mRNA, leading to the degradation and/or translational repression of target genes (77). miRNAs act as regulators in stem cell proliferation, differentiation, apoptosis, and metabolism (78, 79). In this way, miRNAs act as a switch of gene networks, either as an oncogene or as a tumor suppressor gene, and these miRNAs have quickly become an important class of regulatory genes controlling developmental and disease processes. In contrast to transcription factors and molecular signaling pathways, miRNAs involved in stem cell lineage determination have only just started to be studied. An increasing number of miRNAs have been found to be implicated in BCSCs to regulate fate decisions.

Interestingly, miRNAs with micro size but macro function are known to have profound effects on maintaining and regulating the behavior of BCSCs by specifically targeting relevant TFs and oncogenic signaling pathways and play an important role in breast cancer initiation and prognosis. miRNAs serve as oncogenes as well as tumor suppressors. Based on the current findings, we will focus on describing the regulatory role and underlying mechanisms of miRNAs management of BSCS self-renewal, differentiation, metastasis, EMT, drug resistance and recurrence as potential links to breast cancer pathogenesis. Analysis of 11 surgically resected breast cancer patient samples revealed differentially expressed miRNAs in human BCSCs versus nontumorigenic cells (NTG cells) (80). Three clusters of miRNAs, including miRNA-200c-141, miR-200b-200a-429 and miR-183-96-182 cluster, were consistently downregulated in human BCSCs (80). The miR‐200 family maintains the stemness of BCSCs and is able to target the EMT-associated transcription factor ZEB1, thereby up-regulating E-cadherin, the expression of which is reduced and its inhibitory effect on EMT is diminished (80–83). Furthermore, other miRNAs, including let-7, miR-27b and miR-185-3p, were differentially expressed in BCSCs and NTG cells (84–86). It was recently shown that in BCSCs E2F1 binds to the Nanog gene to promote its transcription and that miR-185-3p can target E2F1 leading to a reduction in its expression, thereby inhibiting the stemness of BCSCs (86). Similarly, miR-378a-3p and miR-378d can activate the WNT and NOTCH pathways through targeted inhibition of DKK3 and NUMB, leading to doxorubicin (DOX) and paclitaxel (PTX) resistance (87). Taken together, these discoveries show that miRNAs are instrumental in determining the fate of BCSCs by targeting key coding TFs and related signaling pathways.

## Long noncoding RNAs

LncRNAs are ncRNAs with transcripts longer than 200 nucleotides and little or no protein-coding function. They regulate gene expression and are involved in biological processes such as apoptosis, metastasis, stemness maintenance, proliferation, differentiation, metabolism and drug resistance. LncRNAs can repress or activate gene expression through a variety of mechanisms and exhibit specific expression patterns in different cell and tissue types, respond to different stimuli, and regulate cell fate (88). In the last decade, researchers have shown great interest in the role of LncRNAs in CSC lineage Commitment and differentiation.

LncRNAs influence cell growth, apoptosis and tumor metastasis by participating in epigenetic, transcriptional or post-transcriptional gene regulation. Brown and colleagues summarized the LncRNAs in BCSCs and revealed that a series of BCSC-associated LncRNAs were enriched in TNBC (89). Notably, LncRNAs show differential expression in BCSCs versus non-BCSCs. LncRNA lnc030, which is highly expressed in BCSCs, is able to stabilize squalene epoxidase (SQLE) mRNA cooperating with poly(rC) binding protein 2 (PCBP2) and promote cholesterol synthesis, thereby activating PI3K/Akt to amplify the stemness properties of BCSCs (90). Likewise, high expression of LncRNA-ROPM can increase the stability of PLA2G16 mRNA, thereby promoting phospholipid metabolism and activating PI3K/AKT signaling (48). In addition, other LncRNAs that are upregulated in BCSCs, such as LncRNA-ROR, LncRNA-HOTAIR, LncRNA-HAL, LncRNA-Hh (91) and LncRNA-PVT1 (92), are able to induce EMT, consequently increasing the percentage of BCSC population and stemness. As LncRNAs research progresses, more and more LncRNAs will be demonstrated in the regulation of BCSCs. LncRNAs is a novel regulator of BCSCs by regulating mRNAs, miRNAs and other LncRNAs and will improve the understanding of new molecular regulation of BCSCs.

## Tumor microenvironment

The tumor microenvironment (TME) plays a pivotal function in several steps of tumorigenesis and progression, including drug resistance, immune escape and distant metastasis. The microenvironment regulates the biological behavior of BCSCs through direct contact or ECM and paracrine factors (93). The microenvironment provides fuel and a proper niche for BCSCs, highly regulates their fate, protects them from genotoxicity and improves their tolerance to treatment. Reciprocally, BCSCs are able to influence the TME while adapting to changes in the TME. The TME mainly consists of surrounding normal tissue cells, tumor stroma and microvessels. For example, tumor cells can release immune inhibitory cytokines to evade detection by immune cells in TEM, resulting in immune escape (94). Concurrently, the TME provides the driving force for BCSC plasticity, inducing angiogenesis and recruitment of immune and stromal cells, which in turn accelerates tumor invasion and metastasis.

Stromal cells, such as cancer-associated fibroblasts (CAFs), are verified that affect BCSC activity through the cell-cell interactions, the secretion growth factors, cytokines, chemokines, and the remodeling of the ECM (95). These secreted factors are involved in a variety of regulatory roles for cells in TME and tumor cells. In particular, CAFs, a major component of the stroma, have been shown to support CSC function by secreting cytokines such as IL-6, IL-8 and IL-1β, activating signaling pathways, and promoting BCSC stemness and plasticity (96). The origin of CAFs is now thought to be multiple, including transference of resident fibroblasts (97), transdifferentiation of perivascular cells (97), differentiation of mesenchymal stem cells (MSCs) and EMT. CAFs haven been found to be able to secrete periostin, which in turn recruits Wnt ligands, activates intracellular Wnt signaling in BCSCs, remodels the ECM, establishes a nascent stromal niche and creates the conditions for metastatic colonization of BCSCs (98). Similarly, CAFs also secrete FGF5, which promotes fibronectin collagen formation and remodels the ECM, resulting in the induction of a reversible BCSC phenotype preferentially at the tumor-stromal interface (49).

In addition, CAFs are involved in regulating the biological behavior of BCSCs through their association with other signaling pathways. Activation of WNT/β-catenin and HGF/Met signaling in the mammary gland tumors accelerates the secretion of the Hedgehog ligand SHH in BCSCs, which regulates CAFs *via* a paracrine pathway, and in turn CAFs further secrete factors (99). Accordingly, the Hedgehog inhibitor vismodegib was able to reduce the activity of fibroblasts and breast cancer-forming cells, mechanistically indicating that Hedgehog signaling to CAFs is a potential mediator of CSC plasticity and an intriguing new therapeutic target in breast cancer (49). MSCs and CAFs express high levels of PEAK1 protein in a PEAK1-dependent manner, which activates p-AKT, enhancing tumorigenesis (100). In addition, when MSCs were co-cultured with breast cancer cells, they were able to induce aberrant expression of microRNAs, such as mir-199a upregulation, providing breast cancer cells with enhanced BCSC properties (101). Collectively, these findings identify a potential mechanism of crosstalk between stromal cells and BCSCs and aberrant signaling pathway perturbations, and therefore the development of targeted inhibitors may offer a novel therapy strategy for the management of breast cancer.

Macrophages are a group of plastic and heterogeneous cells that are involved in the innate immune response as another major component of the TME and are capable of regulating the formation and maintenance of BCSCs through the modulating the M1/M2 phenotype. It has been shown that tumor-associated macrophages (TAMs) can activate Src and NF-κB *via* EphA4, which in turn induce the secretion of a variety of cytokines such as IL-6, IL-8 and GM-CSF, thereby establishing a BCSC niche (102). Consistently, in breast cancer, the reduction of macrophages reduced the number of BCSC population (103). In addition, TME-derived endothelial cells provided Jag1 to neighboring BCSCs, increasing the upregulation of zeb1, which in turn increased VEGFA production by ectopic zeb1, inducing endothelial cells to express jag1 in a paracrine manner (29). Similarly, the cell-cell interaction of BCSCs with CD8+ T lymphocytes in TME can establish immune tolerance, mainly due to the ability of BCSCs highly expressing PD-L1 to bind to the PD-1 receptor on the surface of T cells, which in turn exerts an inhibitory effect and leads to T cell exhaustion (104). In addition, ECM, a major component of TME, is a niche that determines the behavior of BCSCs, such as hydroxylated collagen, hyaluronic acid, integrates the intra-/extra- cellular environment signals and activates multiple signaling pathways leading to BCSC metastatic growth (105, 106).

**Figure 3:**
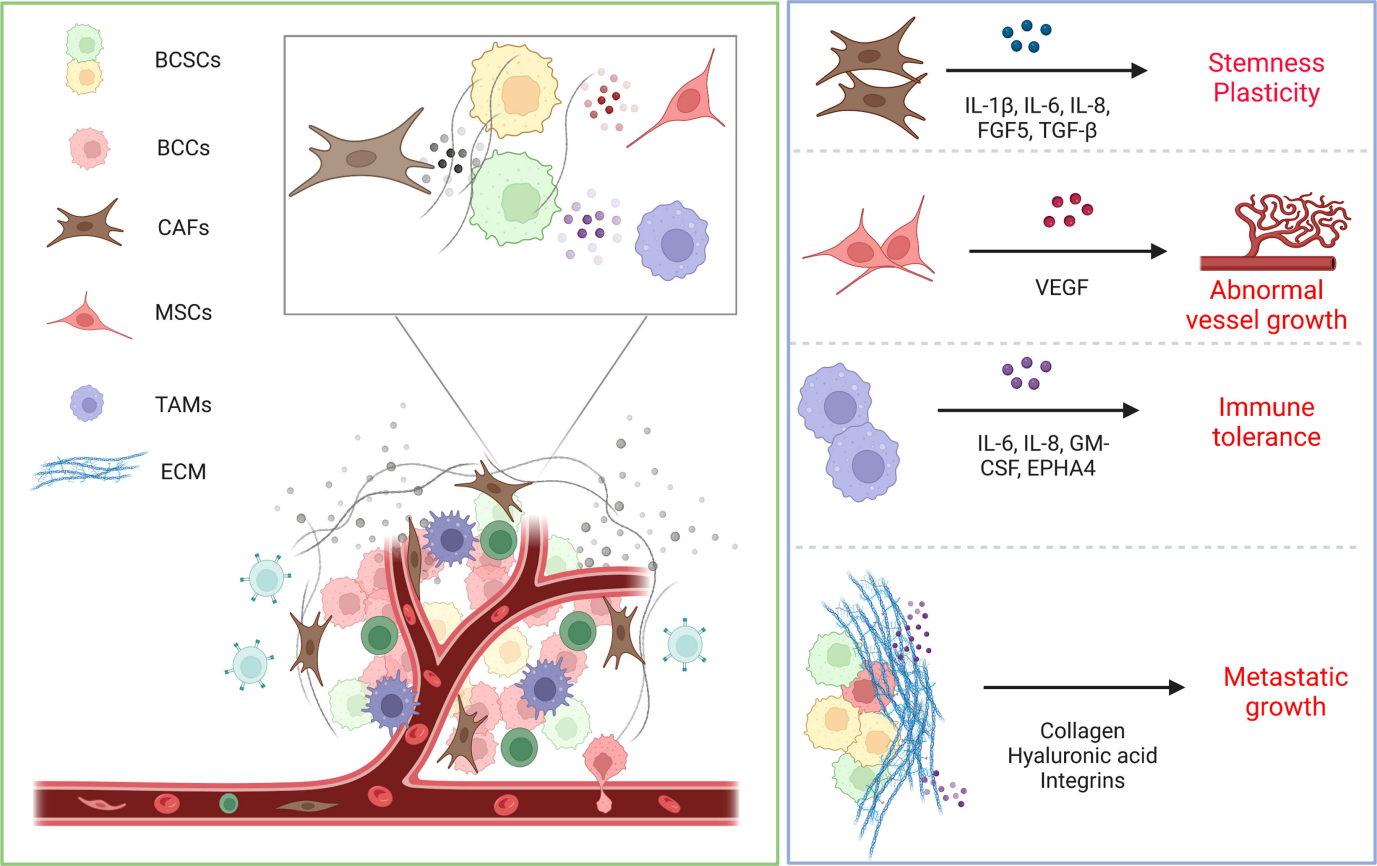
Schematic representation of interactions between TME and BCSCs. The microenvironment regulates the biological behavior of BCSCs through direct contact or ECM and paracrine factors. CAFs secrete cytokines such as IL-6, IL-8 and IL-1β to promote BCSC stemness and plasticity. MSCs secrete VEGF to feed BCSCs, leading to abnormal vessel growth. Macrophages likewise secrete various cytokines that establish the BCSC niche and lead to immune tolerance. ECM offers protection to BCSCs from treatment pressure and safeguards their metastatic growth.

In a nutshell, the TME provides a niche for BCSCs and governs their biological behavior. Importantly, the TME varies markedly between patients, so an exhaustive understanding of the interactivity of the components of the TME on tumor progression is paramount. It has been revealed that TME is potentially of a complex character (107). In parallel, the heterogeneity of TME has been shown to be a potential prognostic factor in identifying different subtypes of breast cancer (108). Building on this, further refinement of breast cancer types and understanding of the specificity of BCSCs offers the potential to accurately predict tumor prognosis and develop new personalized treatment strategies (**Figure 3**).

## Signaling pathways

BCSCs are usually quiescent and are able to transforming their phenotype through EMT, metabolic plasticity, and microenvironment, resulting in limited specific markers. Therefore, more researches have focused on defining the mechanisms of relevant signaling pathways regulating the tumor initiating ability of CSCs. Exhilaratingly, insightful investigations have verified that many key signaling pathways are implicated in modulating the lineage commitment and biological processes of BCSCs.

### Wnt

The Wnt family consists of a large number of secreted glycoproteins with both paracrine and autocrine functions (109). Wnt is participating in many important biological processes (109–111). Wnt ligands bind to the seven transmembrane structural domains of the frizzled receptor, FZD) and LRP5/6 co-receptors and stabilizes β-catenin by preventing its phosphorylation (112). The Wnt pathway plays a key role in BCSC fate.

Abnormal WNT/β-catenin signals are more prevalent in breast cancer, and clinical evidence indicates that increased WNT/β-catenin signals are correlated with higher tumor grade and poorer prognosis (98, 113). In BCSCs, WNT/β-catenin is relevant to stemness, plasticity and microsphere formation (114). Canonical and noncanonical Wnt signaling pathways by promoting the expression CD44 and ALDH1, which in turn increase the stemness of BCSCs. Canonical Wnt signaling through β-catenin stabilization and subsequent nuclear translocation leads to transcriptional activation of β-catenin-TCF/LEF target genes. Inhibition of β-catenin reduces BCSC population, tumor size and resistance to doxorubicin (Dox) in TNBC cells (115). Non-canonical WNT/Ca^2+^ signaling regulates the biological behavior of BCSCs through the activation of RTKs such as ROR1/2 and PI3K/AKT. Just as, Wnt plays an important role in BCSC, so targeting the canonical and/or noncanonical Wnt signaling pathway may be an effective marker for eliminating BCSCs. Recent studies showed that DKK1 inhibited lung metastasis by inhibiting PTGS2-induced macrophage and neutrophil recruitment and thereby antagonizing non-classical WNT/PCP-RAC1-JNK signaling. Conversely, DKK1 promotes bone metastasis by regulating canonical Wnt signaling of osteoblasts (114). These results reveal that amplified Wnt signaling is instrumental in the self-renewal, apoptosis inhibition and metastasis of BCSCs, and therefore inhibition of wnt is essential for the elimination of BCSCs (**Table 2**). A growing number of preclinical researches are treating breast cancer by targeting inhibition of Wnt signaling in BCSCs including OMP-18R5 (Vantictumab) (123), NSC668036 (124) and Pyrvinium pamoate (PP) (122).

**Table 2:** Antagonist of WNT signaling and their effects on BCSCs.

Antagonist	Target	Functional effects	Ref.
PF-06647020	PTK7-ADC	Tumor regressions and outperforming standard-of-care chemotherapy in PDX model	(116)
OMP-18R5 (Vantictumab)	FZD1/2/5/7/8	Synergistic activity with standard-of-care chemotherapeutic agents	(117)
XAV93	Tankyrase 1/2	Combination paclitaxel for TNBC and external carcinogen-induced breast cancer	(118)
LGK974	PORCN	Inhibition of MMTV-Wnt1-driven mechanistic breast cancer models in mice and rats	(119)
Celecoxib	Wnt/β-catenin	Inhibition of the Wnt/β-catenin pathway to eradicate BCSCs	(120)
Sulforaphane	Wnt/β-catenin	Inhibition of BCSCs and the Wnt/β-catenin self-renewal pathways	(121)
Pyrvinium pamoate	Unknown	Inhibition of stemness regulator expression and tumor regressions in NOD/SCID mice	(122)
IONP	Wnt/β-catenin	Inhibition the expression of Wnt/β-catenin, CD44 and uPAR	(115)

### Notch

The Notch signaling pathway enables ligand-receptor interactions through direct cell-to-cell contact. In mammals, the Notch signaling pathway involves the Notch receptor (Notch1-4) and Notch ligand, which is divided into two classes (Jagged1-2 and Delta-like 1,3,4) that differ due to the presence of a cysteine-rich structural domain in the Jagged ligand (125). Notch receptors are activated by ligands on their neighboring cells, which trigger signals regulating various cellular differentiation processes.

Notch signaling plays a variety of roles in cancer, including oncogenesis, carcinogenesis or both. Concurrently, notch pathway is associated with many aspects of cancer biology, including metabolism, metastasis, drug resistance and the maintenance of CSCs. Multiple discoveries have confirmed that Notch signaling is associated with CSC activity in various forms of breast cancer. A meta-analysis of tumor molecular landscapes and several pathological studies have shown that Notch1 activity is associated with the risk of recurrence in ER^+^ breast cancer (126). Endocrine resistant BCSCs, most of which are Notch4-dependent, are a major factor in tumor recurrence and death (127). Interestingly, unlike Notch4, which is predominantly located in the basal cell population, Notch1 is predominantly expressed in the luminal cells of normal breast epithelium, indirectly suggesting that both may play this specific role in different subpopulations of BCSCs (127, 128). In patients with trastuzumab-resistant and HER2^+^ breast cancer, Notch1 expression was associated with poorer prognosis (129). Under the circumstances, abrogation of Notch1 expression resulted in a significant reduction of cancer proliferation *in vivo* (130). In particular, Notch3 was capable to act as a mediator of PD-L1 overexpression in BCSCs, activating mTOR and maintaining the self-renewal and invasive capacity of BCSCs (131). What’s more, it has been reported that Notch3 does effectively downregulate Notch1 signaling by repressing the expression of the downstream genes Hes1 and Hes5 (132). Interestingly, In ER^-^ human breast cancer samples, survival advantage of Notch2^High^ over Notch2^Low^ patients in primary and bone metastatic breast cancer (133). Taken together, these observations suggest a common theme: deciphering the variation in the expression of Notch family members in different breast cancer types is necessary to develop effective treatments for the eradication of BCSCs.

### Eph

Eph receptors are the largest family of RTKs in mammals and are activated by membrane-linked Ephrin ligands (134–136). The Eph receptor and its Ephrin ligand have been implicated as cell-cell communication complexes that influence the behavior of epithelial cells (137). The function of the Eph/Ephrin in the initiation of breast cancer has been analyzed in detail. In the Eph/ephrin system, chromosomal abnormalities, gene methylation, and alterations in transcription regulators induce dysregulation of the Eph/ephrin expression and tumorigenesis (136). It was demonstrated that EPHB6, an intrinsically catalytically inactive member of the Eph group, partially inhibits EMT, synergistically activates RAS-ERK signaling and promotes the expression of OCT4 in BCSCs, thus exhibiting higher stemness (138). PF-06647263 was a humanized monoclonal antibody that selected Ephrin-A4 as a pharmacological target to inhibit the activity of Ephrin-A4, which was highly expressed in BCSCs, in order to alleviate the clinical symptoms of TNBC (139). Importantly, understanding the complexity of the Eph/Ephrin system will help to elucidate the mechanisms of breast cancer.

### Hedgehogs

Hedgehogs signaling includes SHH, IHH and DHH. The precursors can be cleaved to produce an active 19kd N-terminal fragment which binds to the membrane protein Patched gene (Ptc) and Smoothened gene (Smo). As Hedgehog genes are linked, Smo is released, leading to the activation of transcription factors (Gli1-3). In BCSCs, tetraspanin-8 (TSPAN8) was significantly upregulated, recruiting the deubiquitinating enzyme ATXN3 to inhibit the degradation of the SHH/PTCH1 complex, leading to SMO translocation to cilia, causing resistance to chemotherapeutic agents in CSCs and enhancing tumorigenesis in mice (140). Dehydrocholesterol reductase (DHCR24), a key enzyme in cholesterol synthesis, could promote breast cancer development by enhancing the Hedgehog and BCSC populations (141). While, Neuropilin-2 (NRP2) had the ability to activate Gli-1 and α6β1 integrins to induce BCSC initiation (142). Further in depth, Gli-1 and α6β1 integrins mediated the self-renewal and progression of BCSC by promoting angiogenesis and triggering focal adhesion kinase (FAK) signaling, respectively (143, 144). Consequently, targeting the SHH, α6β1, TSPAN8, and FAK can represent an attractive strategy for breast cancer treatment. Curcumin, a polyphenolic compound from the rhizome of Curcuma longa, has been reported to inhibit the proliferation and metastasis of TNBC cells, EMT and BCSC characteristics *via* the Hedgehog/Gli1 pathway (145). Similarly, genistein reduced the population of BCSCs by inhibiting Hedgehog (146). In summary, the search for integrated interventions in Hedgehog signaling and targeted inhibition of BCSC biological behavior could provide a new direction for breast cancer treatment.

### PI3K/AKT

PI3K is an intracellular phosphatidylinositol kinase (147). AKT is composed of three main isoforms (AKT1-3), which are key effectors of PI3K and can be directly activated by PI3K (148). PI3K/AKT is involved in regulating BCSC self-renewal, EMT and invasion (149, 150). PI3K/AKT also induced the of activation WNT signaling, which in turn increased the stemness and metastasis of BCSCs. HER2 dysregulation leads to aberrant activation of (PI3K)-Akt and/or WNT signaling and enhanced activity of the BCSC population, resulting in trastuzumab treatment resistance (151). Reciprocally, the role of the HER2 signaling in BCSCs can be enhanced by the PI3K/Akt pathway (152). Therefore, an open-label phase II study demonstrated that trastuzumab and lapatinib, which targeted HER2, inhibited the expression of FOXO, STAT5 and PI3K/AKT and suppress BCSC subpopulations (153).

Mammalian target of rapamycin (mTOR) is a serine/threonine kinase consisting mainly of two distinct protein complexes, mTORC1 and mTORC2, which are key target genes downstream of AKT (154). Activation of PI3K promotes activation of mTORC1 and mTORC2, while the mTOR activity is frequently upregulated in human cancers (155). What’s more, the mTOR pathway is generally considered to be over-activated in CSCs. The inhibitory effect of some mTOR inhibitors on CSCs has been demonstrated (156). Rapamycin, everolimus and PF-04691502 inhibit tamoxifen-induced activation of BCSCs (157). Inhibition of mTOR restores AKT/mTOR-induced resistance to radiotherapy in BCSCs (158). Although mTOR has a role in suppressing BCSCs, a study showed that treatment of TNBC cells with mTOR inhibitors upregulated FGF1-FGFR-Notch1 signaling, leading to an increase in BCSC population (159). In this case, combined blockade of FGFR or Notch1 may prevent resistance to mTORC1/2 inhibitors by eliminating BCSCs (160). Mechanistically, adaptation or resistance to mTOR inhibition in BCSCs is manifested mainly by transcriptional reprogramming of the EVI1 and SOX9 to upregulate REHB and RAPTOR and metastasis-associated mediators (FSCN1 and SPARC) (161). Corporately, a link between PI3K-Akt-mTOR and BCSCs is evident.

## Intertwining of signaling pathways in BCSCs

**Figure 4:**
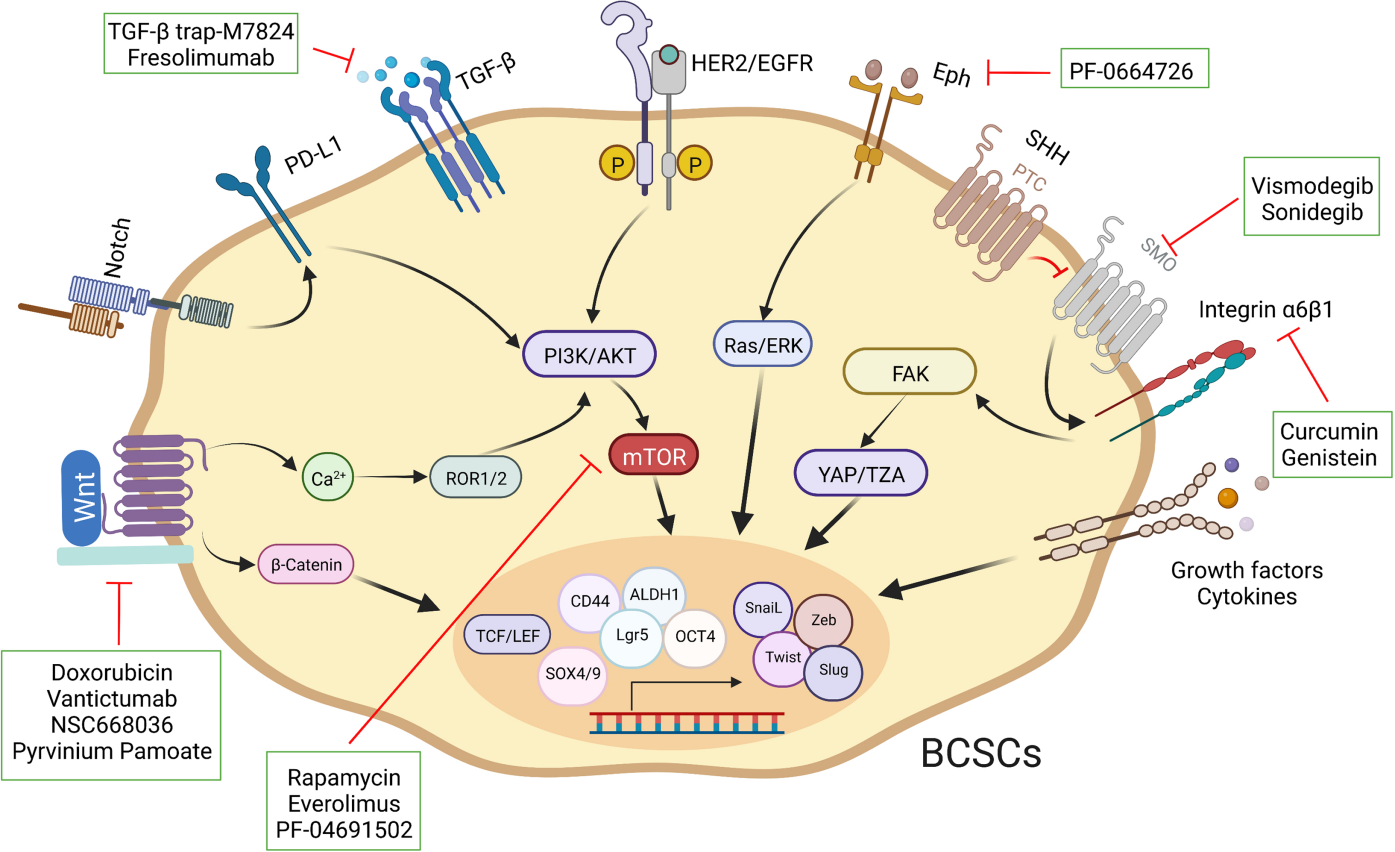
Schematic representation of different strategies used to target BCSCs. Specific pathways have been implicated in the fate of BCSCs. A select set of inhibitors have been developed to inhibit specific pathways.

As described previously, these intricate signal transduction pathways are not linear. The crosstalk among multiple pathways is also common in breast cancer, for instance, a discovery has revealed that Syndecan-1 promoted the activation of IL-6/STAT3 and EGFR *via* Notch to regulate inflammation and phenotype of BCSCs (162). The Hippo transducer TAZ confers BCSC-related features, including self-renew and tumor-initiation capacities, through MET (42). FAK can regulate YAP/TAZ activation (163). Aberrant regulation of signaling pathways, such as ERα, Notch and Hedgehog, can lead to abnormal activation of Hippo, resulting in BCSC fate perturbations (164–166). The cumulative effect of aberrant regulation of these pathways in breast cancer maintains and enhances the characteristics of BCSCs, ultimately culminating in malignant tumor progression. Consequently, a thorough insight into the perturbations of different pathways in individual patients is necessary to optimize personalized therapeutic strategies. Importantly, fully assessing the characteristics and subpopulation distribution of BCSCs and developing novel vehicles to eliminate them (**Figure 4**).

## Therapeutic strategies to target BCSCs: an adventurous voyage

From a clinical perspective, deciphering the relevance of BCSCs in therapy resistance, including chemotherapy, radiotherapy, immunotherapy and endocrinotherapy, is one of the major challenges in the clinical translation of anti-CSC therapies. Actually, CSCs are involved in tumor recurrence, metastasis and drug resistance, therefore targeting CSCs may be helpful and complementary to the treatment of breast cancer, combating concerns about safety and treatment failure. Excitingly, researchers have recently explored targeted therapeutic strategies for BCSC quiescence, maintenance pathways and specific markers.

The availability of BCSC-specific markers has facilitated researchers to effectively identify them, and commonly markers used to isolate BCSC include CD44^+^CD24^-^, CD133 and ALDH1. The high expression of these phenotypically and functionally significant markers in BCSCs compared to normal tissue could allow novel drugs to identify and block relevant BCSC signaling pathways, making them more susceptible to elimination by therapeutic strategies. CD44 is a cell surface receptor that binds to its ligand hyaluronic acid (HA) and that activates a variety of intracellular signals, and the interaction between them is used as a drug target. A study has demonstrated that lapatinib nanocrystals coated with HA have better therapeutic efficacy than uncoated HA in TNBC (167). Comparably, CD133, a membrane glycoprotein, has a demonstrated association with tumor resistance and recurrence. Polymeric nanoparticles loaded with paclitaxel targeting CD133 can markedly reinforce CD133^+^ cell internalization while significantly suppressing tumor regrowth in a xenograft model (168). Going further, conjugation anti-CD133 mAbs with saporin causes CD133^+^ BCSC proliferation arrest followed by cell death (169). However, unlike the traditional membrane proteins, ALDH1 is an enzyme with an activity that is intimately associated with the ability of BCSCs to self-renewal. Therefore, targeting ALDH1 is an effective therapeutic agent to eliminate BCSCs.

In fact, the surface phenotype of BCSCs is constantly in flux during cancer progression, differentiating or evolving into different cancer cells and thus obtaining distinct phenotypic recurrences. As a consequence, this will be the most prominent challenge in the design of targeted BCSC therapeutic interventions. Mechanistically, BCSCs undergo cell fate shifts in response to therapeutic pressure or metastasis, leading to malignant progression, which is mainly driven by their inherent genomic and epigenetic instability. Consequently, the strategy applied in clinical trials should take adequate consideration of the comprehensive range of elements leading to selective cell fate decisions, including the tumor microenvironment, intratumor heterogeneous cells and signaling cascades. The TME supports the self-renewal and differentiation of BCSCs, providing a niche to regulate their cell fate in the form of secreted factors and intercellular communication. The xenograft NOD/SCID mice model demonstrated that by affecting the expression of IL-6 in the local microenvironment of BCSCs, it was possible to regain ER expression and subsequently CD133^hi^ cells were able to respond to hormone therapy (170). Inevitably, the damage to a single local microenvironment established through these animal models alone cannot fully replicate the reality of human breast cancer progression, but these adventurous research methods provide an important theoretical and temporal basis for extending preclinical studies. With technological advances, methods such as primary cell culture, organoid culture and microfluidic 3D biomimetic model allow for an improved mimicking of the normal tissue microenvironment, thus providing a new voyage to target the variable traits of BCSCs (171, 172).

Of vital note, signaling pathways are one of the key factors regulating the maintenance and evolution of BCSCs, and therefore targeting these key signals has proven to be an invaluable vehicle for the elimination of BCSCs. The major signaling pathways include Wnt, Notch, Eph, Hedgehogs and PI3K/AKT, which often interact with together in breast cancer stem cells during the development of breast cancer. Equally excitingly, with intensive research into cellular immunity, an additional option for oncology treatments has been developed with novel anti-BCSC immunotherapies such as immunologic checkpoint blocking or CAR-T cell therapies. PD-L1 is detected in 20% of TNBC (173). Deletion of RBMS1 expression by specific shRNA activates PD-L1 immune checkpoint receptor blockade to promote anti-tumor immunity in TNBC (174). In a phase I clinical study of 54 TNBC patients, Atezolizumab showed an objective response rate of 19% as an inhibitor of PD-L1 (175). For CAR-T cell therapy, TEM8 (176) and NKG2D (177) have been used for BCSC-targeted immunotherapy. Collaboratively, these discoveries shed new perspective on the preparation of clinically feasible therapeutic strategies for targeting BCSCs (**Table 3**).

**Table 3:** Targeting BCSCs with different agents in clinical trials.

Agents	Target	Sample size	Phase	Status	NCT Number
Bevacizumab	ALDH1	75	II	Completed	NCT01190345
MK-0752	Notch	30	I/II	Completed	NCT00645333
LDE225	Hh	30	I	Completed	NCT01954355
AZD8055	PI3K	64	I	Completed	NCT00731263
OMP-54F28	Wnt/β-catenin	26	I	Completed	NCT01608867
Reparixin	CXCR-1	33	I	Completed	NCT02001974
LY2157299	TGFBR1	12	I	Completed	NCT01722825
Lutetium Lu 177 Dotatate	SSTR2	10	II	Not yet recruiting	NCT04529044
GSK3326595	PRMT5	60	II	Not yet recruiting	NCT04676516

## Conclusion and prospect

To date, we recognize that BCSCs are a small population of cancer cells with self-renewal and differentiation potential that are involved in mediating tumor heterogeneity, recurrence, metastasis and treatment resistance. There is current research indicating that BCSCs are an attractive target for tackling resistance and recurrence in the clinical therapy of breast cancer. Fortunately, BCSCs have the expression of their own specific markers that can provide post-therapeutic local biopsies with timely information on treatable targets for the remaining tumor tissue on the basis of variable biomarkers, thus allowing the selection of targets for the use of personalized and precise second-line therapy (178, 179). Especially, it is the introduction of the breast cancer stem cell concept, which focuses on biomarkers of BCSCs in the post-treatment period, that offers a new alternative to combating tumor recurrence. However, further attention needs to be given to the fact that normal stem cells in the tissue may also express the overlapping biomarkers and signaling pathways as BCSCs. This therefore requires that the possible side effects of targeting BCSCs for the treatment of breast cancer be fully considered, which in turn requires the rigorous elaboration of identity markers and signaling patterns that are specific or even unique to the targeted BCSCs. Meanwhile, BCSCs tend to have quiescent properties during response therapy, so therapeutic strategies to inhibit tumor progression do not fully prove to be due to the efficacy of targeted inhibition of BCSCs. In addition, BCSCs exist in a specific niche surrounded by heterogeneous cells such as TAMs, MSCs and CAFs that maintain their long-term survival. However, most current researches deficient a microenvironment have used isolated BCSCs and the relationship between BCSCs and their niches is currently ambiguous. Finally, it is undeniable that the immunodeficient animal models lacking adaptive immunity used in the current studies on BCSCs are not capable of recapitulating the biological complexity of tumors in the clinic (180). Collectively, there are still many obstacles to cross in achieving efficient and safe elimination of BCSCs.

In conclusion, the discovery of BCSCs has well revealed that individual cancer cells from the same tumor exhibit essential heterogeneity in terms of mutations, transcriptional programs, immune characteristics and functional properties. Indeed, BCSCs exist in a dynamic state, with multiple pools in individual tumors, so combining multiple treatment strategies to eradicate the pools of therapy-resistant BCSCs on the top of the heterogeneity is clinically important for preventing cancer recurrence. Deeply, cellular plasticity that mediates stemness, fueling cancer heterogeneity and responding to therapeutic pressure, further leading to the limitations of anti-CSC therapeutic strategies. Importantly, the BCSC concept not only has broad and profound implications for our understanding of cancer origins and progression, but also has significant clinical value for the design of more effective and personalized treatment options in the future. Therefore, a combination of conventional cytotoxic drugs, immunotherapy agents, endocrine therapy and eradication of BCSC therapy is a future direction of great significance for improving the clinical prognosis of breast cancer.

## Author contributions

HX and LZ contributed to the conception of the study. HX and FZ were responsible for the collection and assembly of data. XG and QZ were responsible for literature search. All authors were involved in the writing and final approval of the manuscript.

## Acknowledgments

We are grateful to the BioRender.com platform for providing online drawing tools (Agreement number of **Figures 1**-**4**: SG24652NWG, AP241A6DG7, JE241A6DHK, KU241A6DJM).

## Conflict of interest

The authors declare that the research was conducted in the absence of any commercial or financial relationships that could be construed as a potential conflict of interest.

## Publisher’s note

All claims expressed in this article are solely those of the authors and do not necessarily represent those of their affiliated organizations, or those of the publisher, the editors and the reviewers. Any product that may be evaluated in this article, or claim that may be made by its manufacturer, is not guaranteed or endorsed by the publisher.
